# A System of Practical Medicine by American Authors

**Published:** 1886-10

**Authors:** 


A SYSTEM OF PRACTICAL MEDICINE BY AMERICAN AUTHORS. Edited by William Pepper, M. D., LL. D., Provost and Professor of the Theory and Practice of Medicine and of Clinical Medicine in the University of Pennsylvania, assisted by Lewis Starr, M. D., Clinical Professor of Diseases of Children in the Hospital of the University of Pennsylvania. Vol. V. Diseases of the Nervous System. Philadelphia: Lea Brothers & Co. 1886.

The editor, in presenting to the profession the fifth and concluding volume of the "System of Practical Medicine by American Authors," states that the original prospectus of the work was issued in 1881. The first volume was published in January,

1885; the second in May, 1885; the third in September, 1885, and the fourth in February, 1886. The number of articles is 185, written by 99 authors, covering, with indexes, about 5,600 pages, and throughout its whole extent the original purpose has been kept constantly in view, that the practical character of the work should adapt it specially to the needs of the general practitioner.

The great importance of clear information as to the various diseases of the nervous system, based upon the recent investigations in their developments, renders this collection of articles in the form of monographs of much value to the medical profession.

While many practical subjects are treated exhaustively by the several authors, the names of Seguin and Spitzka are the only specialists in this department, which appear among the contributors to this volume devoted to nervous diseases. This may be regarded rather as a recommendation than a detraction, since the reader is furnished with more clinical than theoretical data in the results of their investigations. Without undertaking to comment upon the satisfactory execution of their parts by other individuals who have co-operated in this work, it may be excusable to note especially the papers on "Diseases of the Peripheral Nerves" and on "Vaso-Motor and Trophic Neuroses," as meeting the wants of the profession at the present day.

This volume affords a most excellent resume of the existing knowledge in regard to the diseases of the nervous system, and is a fit conclusion of the entire subject of practical medicine presented in this valuable work.

